# Supplementary material for: Bioinspired Hierarchical Soft Gripper with Hexagonal and Suction Interfaces for Strain-Guided Object Handling
Source: Biomimetics (Basel). 2025 Aug 4;10(8):510. doi: 10.3390/biomimetics10080510 (PMC12383327; doi:10.3390/biomimetics10080510)
Supplement: Supplementary file 1 [file biomimetics-10-00510-s001.zip › biomimetics-3730738-supplementary.pdf]

Supplementary Materials

Bio-inspired Hierarchical Soft Gripper with Hexagonal and suction interfaces for strain-guided object handling

Junho Lee [1], Junwon Jang [1], Taeyoung Chang [1], Yong Jin Jeong [2], Young Hwan Park [1], Jeong Tae Seo [1], and Da Wan Kim [1*]

[1] Department of Electronic Engineering, Korea National University of Transportation, Chungju-si, Chungbuk 27469, Republic of Korea;

[2] Department of Materials science and Engineering, Korea National University of Transportation, Chungju-si, Chungbuk 27469, Republic of Korea;

wnsgh1916@a.ut.ac.kr (J.L.); yjjeong@ut.ac.kr (Y.J.); pyh@ut.ac.kr (Y.P.); jtseo@ut.ac.kr (J.S.); wertt1027@a.ut.ac.kr (J.J.); 2022139@ut.ac.kr (T.C.); dawankim@ut.ac.kr (D.K.)

*Correspondence: dawankim@ut.ac.kr

Supplementary Note

Assembly Strategy for Sensor Integration with MCP3421 and ESP32. Multiplexer control and data acquisition are managed by the ESP32 microcontroller, which is soldered directly to the PCB through a matrix of via-holes precisely matching its pin layout, ensuring both electrical and mechanical stability. The final board layout includes four M3 mounting holes—one at each corner—for secure attachment to robotic arms or test fixtures. The PCB is fabricated on 1.6 mm-thick FR-4 substrate, and all components are assembled using surface-mount technology followed by a reflow soldering process to optimize component alignment and solder quality.

PCB Design Principle for Strain-Gauge Signal Acquisition. A Wheatstone, bridge-based circuit was designed to measure the resistance change of the strain gauge, which operates in the 90–120 Ω range. In its initial balanced state, no current flows across the midpoints of the bridge. Upon mechanical deformation, the resistance of the strain gauge varies, leading to an imbalance in the bridge and producing a measurable voltage difference.

The design objective was to convert these small voltage changes into high-resolution digital signals and to efficiently acquire data from multiple sensor channels. To this end, a commercial analog-to-digital converter (ADC) module with a built-in instrumentation amplifier was used. The output voltage from the bridge was adjusted to fall within 0–250 mV to match the input voltage range of the ADC (2.048 V reference). This adjustment ensured optimal utilization of the ADC's resolution, as the raw output voltage from the strain gauge would otherwise fall below the effective digitization threshold.

The digital voltage was calculated using the following equation:

$$R = \frac{30}{2.048} \times V_{Measured\ Voltage} + 90$$

Here, the coefficient $\frac{30}{2.048}$ acts as a scaling factor that converts the measured voltage from the ADC into the corresponding resistance value. The numerator 30 represents the full measurable resistance range of the strain gauge (from 90 Ω to 120 Ω), and 2.048 V is the ADC's reference voltage. The constant 90 corresponds to the baseline resistance of the strain gauge when no strain is applied, thereby providing an accurate reconstruction of the actual resistance.

Four ADC modules were connected to an I^2C multiplexer (TCA9548A), which enabled sequential sampling of each sensor channel via a single I^2C bus. This system-level configuration goes beyond simple component selection and was designed to optimize signal resolution, bandwidth, multi-channel expansion, and real-time wireless data transfer.

The digitized signals were transmitted to a PC via an ESP-32 microcontroller, which collected the output from the ADC modules and relayed the data wirelessly for storage and analysis.

Strain Sensor Specification. A commercial foil-type strain gauge (model SB (SN) #120-P-*) was used, featuring a nominal resistance of 120 Ω and a gauge factor (GF) of 110 ± 5%. The response time of the sensor was approximately 63 ms. The temperature coefficient of resistance (TCR) and temperature coefficient of gauge factor (TCGF) were both less than 0.15%/°C. The sensor operated at a current below 25 mA and was rated for temperatures up to 80 °C. The strain limit was specified as 6000 με. All specifications were based on manufacturer-provided data.

Supplementary Figures

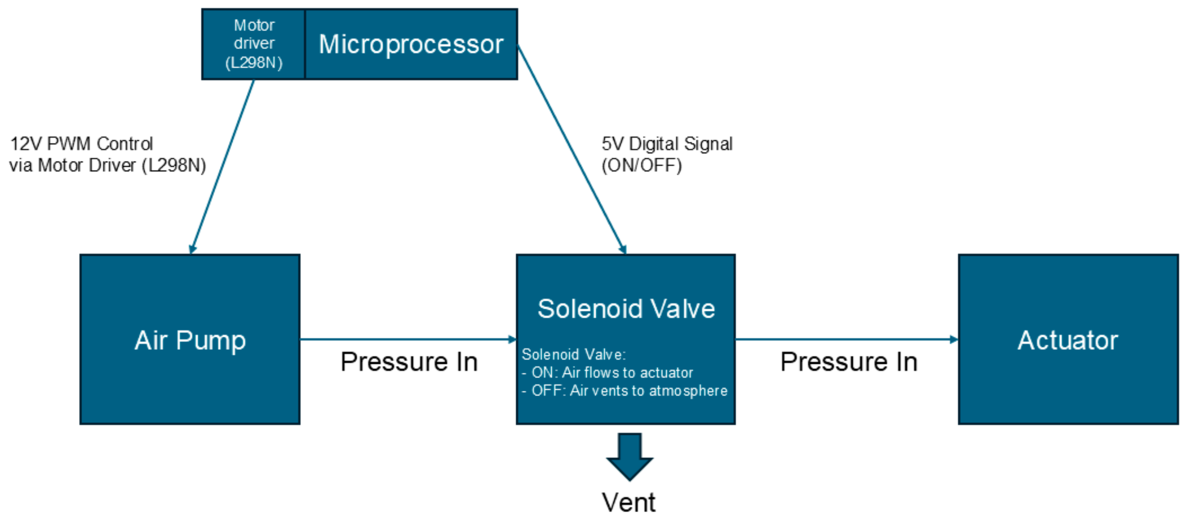

Figure S1. Pneumatic actuation system controlled by a microprocessor via PWM and digital signals.

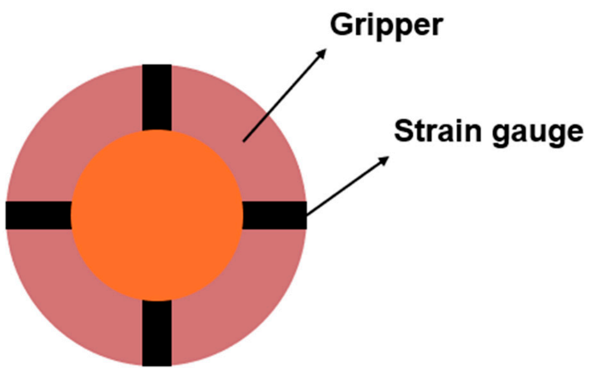

Figure S2. Schematic top view of the gripper showing the layout of the four strain gauges (black), evenly distributed at 90-degree intervals on the upper surface of the infundibulum.

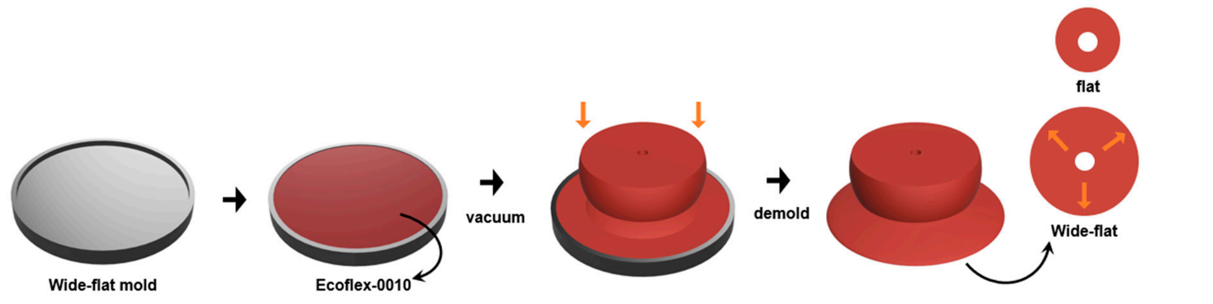

Figure S3. Process for fabricating the wide-flat structure on the bottom surface of the infundibulum.

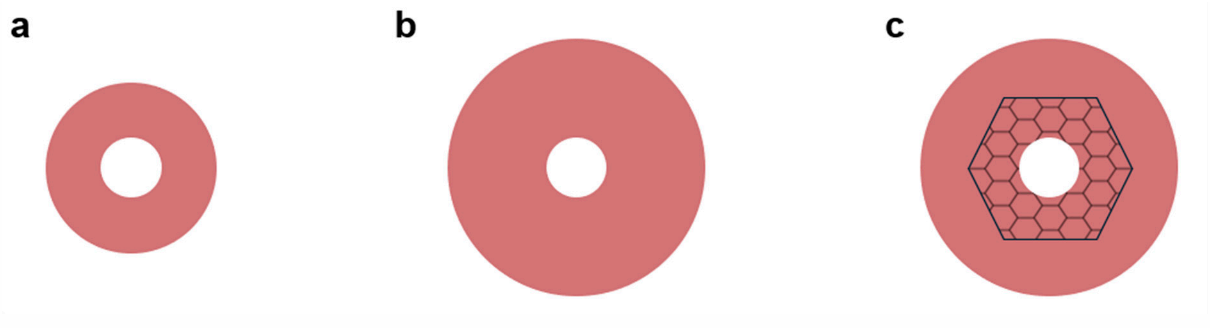

Figure S4. Comparative schematic diagrams of the three infundibulum morphologies: (a) flat (diameter: 33 mm); (b) wide-flat (diameter: 50 mm); and (c) hex-flat (diameter: 50 mm).

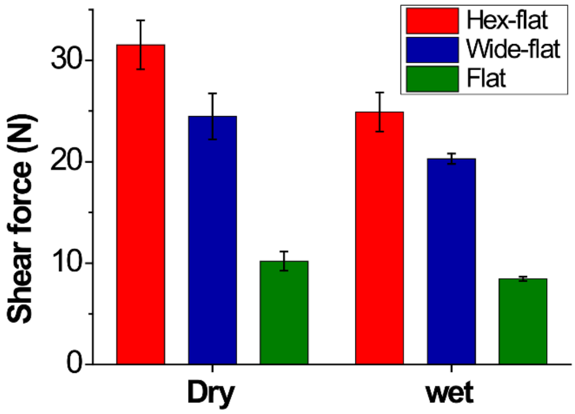

Figure S5. Comparison of shear force performance across different surface configurations (hex-flat, wide-flat, flat) under identical inclined surface conditions.

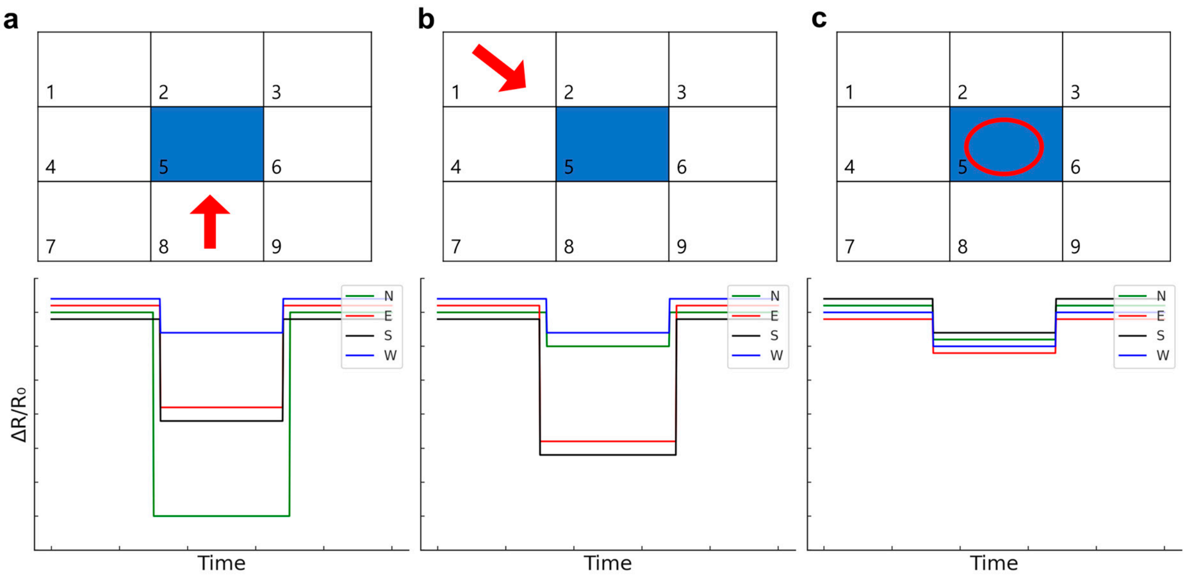

Figure S6. Signal-based inference of directional offset from four strain sensors using rule-based logic. The 3×3 grid represents possible object offset positions during grasping (positions 1–9), with inferred force vectors indicated by arrows. Each positional group is associated with a representative signal response pattern based on the following sensor configuration: N = north sensor (top), S = south sensor (bottom), E = east sensor (right), W = west sensor (left). (a) Single-dominant sensor: one sensor (e.g., W) shows the strongest response, two adjacent sensors (N, S) moderate, and the opposite sensor (E) minimal → offset direction is toward the dominant sensor (e.g., position 6). (b) Two adjacent moderate sensors (e.g., N and E) with weaker responses in the other two → inferred direction lies diagonally between them (e.g., position 3). (c) All four sensors respond at similar levels → contact is inferred to be centered (e.g., position 5). Representative signal illustrations below each case are theoretical plots used to visualize the logic, not direct experimental data. While real signals may vary, these simplified scenarios serve as a practical logic model for estimating directional bias.
